# Evaluation of the safety profile of rotavirus vaccines: a pharmacovigilance analysis on American and European data

**DOI:** 10.1038/s41598-020-70653-3

**Published:** 2020-08-12

**Authors:** Giulia Bonaldo, Roberta Noseda, Alessandro Ceschi, Alberto Vaccheri, Domenico Motola

**Affiliations:** 1 Unit of Pharmacology, Department of Medical and Surgical Sciences, University of Bologna, Via Irnerio 48, 40126 Bologna, Italy; 2 Division of Clinical Pharmacology and Toxicology, Institute of Pharmacological Sciences of Southern Switzerland, Ente Ospedaliero Cantonale, Lugano, Switzerland

**Keywords:** Vaccines, Drug safety, Pharmacology

## Abstract

Rotaviruses (RVs) are the most common cause of severe diarrheal disease. To date two rotavirus oral vaccines are licensed: Rotarix and Rotateq. Our aim was to contribute to the post-marketing evaluation of these vaccines safety profile. We collected all RV vaccines-related reports of Adverse Events Following Immunization (AEFI) in US Vaccine Adverse Events Reporting System (VAERS) and VigiBase between January 2007 and December 2017. A disproportionality analysis using Reporting Odds Ratio (ROR) was performed. A total of 17,750 reports in VAERS and 6,358 in VigiBase were retrieved. In VAERS, 86.2% of the reports concerned RotaTeq, whereas in VigiBase 67.7% of them involved Rotarix. Across the databases, diarrhea (1,672 events in VAERS, 1,961 in VigiBase) and vomiting (1,746 in VAERS, 1,508 in VigiBase) were the most reported AEFIs. Noteworthy, the RV vaccines-intussusception pair showed a ROR greater than 20 in both databases. Some new potential safety signals emerged such as fontanelle bulging, hypotonic-hyporesponsive episode, livedo reticularis, and opisthotonus. Overall, our data show that most of the reported AEFIs are listed in the Summary of Product Characteristics (SPCs). However, there remains the need to investigate the potential safety signals arose from this analysis, in order to complete the description of the AEFIs.

## Introduction

Rotaviruses (RVs) are the most common cause of severe diarrheal disease in young children throughout the world^1^. Diarrheal disease is also among the top ten causes of death worldwide, and in 2016 represented the second major cause of death in low-income countries^2^. To date two rotavirus live attenuated vaccines are licensed. These are the monovalent (RV1) Rotarix (GlaxoSmithKline) and the pentavalent (RV5) RotaTeq (Merk and Co., Inc). In 2018, the World Health Organization (WHO) prequalified others two rotavirus vaccines (Rotavac and Rotasil), not included in this analysis because not available on the market in the period considered. The RV vaccines considered are indicated for the active immunization of infants aged 6–24 weeks (Rotarix) and 6–32 weeks (RotaTeq) for prevention of gastroenteritis due to rotavirus infection^3,4^. The introduction of rotavirus vaccines has decreased the incidence of severe rotavirus gastroenteritis in many countries and rotavirus-associated mortality in several settings^5^. Clinical trials demonstrated the efficacy of both vaccines, with significantly diminished efficacy and effectiveness in low and middle income settings as compared to high-income settings^6–9^. On the basis of these evidences, the WHO recommends the inclusion of rotavirus vaccine in all national immunization programs.

In the last 20 years, some concerns emerged about the safety profile of rotavirus vaccines. In 1999 in the United States, the first rotavirus vaccine, Rotashield, was withdrawn 9 months after approval because of a temporal association with intussusception in vaccinated infants^10–12^. Intussusception is the invagination of one segment of the intestine within a more distal segment and, if untreated, it may be fatal^13^.

After Rotashield withdrawal, great attention has been deserved for post-marketing safety studies focused on the risk of intussusception^14–16^. With this regards, in order to increase the quality of safety data on rotavirus vaccines and facilitate a global safety assessment, the WHO Global Advisory Committee on Vaccine Safety (GACVS) together with other expert groups, recommended a standardized post-marketing surveillance approach. The rationale is to enhance the quality of safety data available to allow a correct assessment of the safety of rotavirus vaccination worldwide^17^.

Our aim was to contribute to the post-marketing evaluation of rotavirus vaccines safety profiles through the analysis of spontaneously reported cases of suspected Adverse Events Following Immunization (AEFI), gathered in the US Vaccine Adverse Events Reporting System (VAERS) and in VigiBase, the WHO global database of Individual Case Safety Reports (ICSRs).

## Methods

### Study population and design

Data was retrieved from the two main pharmacovigilance deduplicated databases worldwide: VAERS and VigiBase. VAERS is the national US vaccine safety surveillance database of Adverse Events Following Immunization. It was established in 1990 and it is co-administered by the Centers for Disease Control and Prevention (CDC) and the Food and Drug Administration (FDA)^18^. VigiBase is the WHO Global Individual Case Safety Reports (ICSRs) database, set up in 1968 by the Uppsala Monitoring Centre; it collects safety reports of suspected Adverse Drug Reactions (ADRs) for drugs and Adverse Events Following Immunization (AEFI) for vaccines from the countries participating in the WHO Program for International Drug Monitoring (PIDM)^19^. Both represent passive pharmacovigilance tools that allow the collection of spontaneous reports of AEFI by different types of reporters (e.g. physicians, marketing authorization holders, patients and others). Spontaneous reporting systems are extremely important because they provide the highest volume of information at the lowest maintenance cost^20^. Although spontaneous reporting systems do not allow establishing a causal association between the suspected vaccine and the reported adverse event(s), they enable the detection of unusual or unexpected patterns of adverse event reporting.

VAERS and VigiBase contain similar information, which include: patient age, sex, medical history and concomitant therapies; country/state of primary source; vaccine characteristics (e.g. type, name, manufacturer, route of administration, batch number or booster) and adverse event features (e.g. seriousness, onset date, outcome). Symptoms are coded using the Medical Dictionary for Regulatory Activities (MedDRA), a highly specific standardized medical terminology that facilitates international sharing of regulatory information^21^. One or more symptoms can be reported for each report.

For the purpose of the present research, we analyzed safety reports gathered in VAERS from January 01st, 2007 to December 31st, 2017, related to the monovalent RV vaccine (RV1) Rotarix and to the pentavalent RV vaccine (RV5) RotaTeq. We applied the same approach to query VigiBase from which we retrieved exclusively European safety reports.

The CDC Wonder online computer interface^22^ was used to manage VAERS data. This database assists in the analysis of public health data through specific queries. We collected all safety reports related to Rotarix and RotaTeq without filtering for age, sex, seriousness or type of reporter, in order to have a complete overview of all the reported AEFI for these vaccines regardless of other factors.

### Data mining

All the safety reports related to RV vaccines were analyzed. Data was categorized per vaccine type, age, sex, seriousness, and year of reporting. The two databases were analyzed separately. A comparative analysis was performed as follows: all the RV vaccines vs. other vaccines, both in VAERS and VigiBase. The analysis was performed using the Reporting Odds Ratio with 95% confidence interval and *p* value ≤ 0.05, as statistical parameter to evaluate vaccine—event pairs distribution. This type of analysis makes use of 2 × 2 contingency table for compared a specific vaccine—reaction frequency to reference distributions of other vaccines from the whole database. If ROR is < 1, there is not disproportionality and the distribution of the events following immunization is the same across vaccines; on the other side if ROR is > 1 there is an increased frequency for the vaccine—event pair considered^24^. For the most frequent events, it was evaluated whether or not they were listed in the Summary of Product Characteristics (SPCs) of the corresponding vaccine made available by the European Medicines Agency (EMA) or the US Food and Drug Administration (FDA).

## Results

### Descriptive analysis of VAERS and Vigibase

During the study period, we retrieved a total number of 17,750 Individual Case Safety Reports (ICSRs) referred to RV vaccines in VAERS and 6,358 in Vigibase (only European cases).

**Figure 1:**
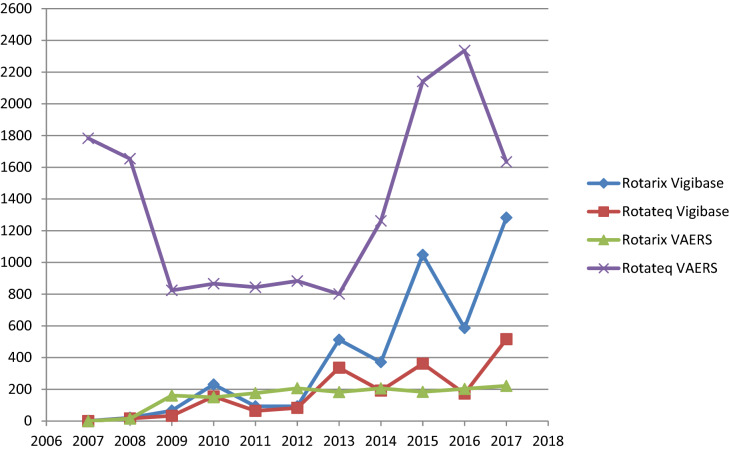
Reporting trends by year and vaccine in VAERS and Vigibase.

Of these 9.8% concerned Rotarix, 86.2% RotaTeq and the 4.0% has no brand name reported in VAERS. In Vigibase the 67.7% was reported for Rotarix, 30.3% for RotaTeq and the 2.0% was without the brand name. Figure 1 shows the temporal reporting trends in the period 2007–2017 for the safety reports related to RV vaccines and gathered in the two pharmacovigilance databases. In VAERS, safety reports concerning RotaTeq were most frequently reported whereas, in VigiBase, the most frequently reported were those about Rotarix. Table 1 shows the classification of RV vaccine safety reports by age and sex in both databases. The majority of safety reports involved children from 0 to 11 months (72% in VAERS, 78% in VigiBase). The proportion of safety reports in the 1–5 years age class was higher in VigiBase (11%) as compared to VAERS (1%). In VAERS, serious safety reports were 3,020 out of 17,750 (17.0%), and in VigiBase 2,508 out of 6,358 (39.4%).

**Table 1:** Characteristics of rotavirus vaccine safety reports retrieved from VAERS and VigiBase.

Age	VigiBase				VAERS			
	Subtotal (%)	M (%)	F (%)	U (%)	Subtotal (%)	M (%)	F (%)	U (%)
< 6 months	4,588 (72.2%)	2,343 (36.9%)	2065 (32.5%)	180 (2.8%)	9,784 (55.1%)	4,708 (26.5%)	4,203 (23.7%)	873 (4.9%)
6–11 months	392 (6.2%)	196 (3.1%)	154 (2.4%)	42 (0.7%)	2,927 (16.5%)	1,299 (7.3%)	1,197 (6.7%)	431 (2.4%)
1–2 years	423 (6.7%)	233 (3.7%)	168 (2.6%)	22 (0.3%)	147 (0.8%)	66 (0.4%)	47 (0.3%)	34 (0.2%)
3–5 years	264 (4.2%)	117 (1.8%)	142 (2.2%)	5 (0.1%)	31 (0.2%)	12 (0.1%)	13 (0.1%)	6 (0.0%)
6–17 years	12 (0.2%)	4 (0.1%)	8 (0.1%)	0 (0.0%)	18 (0.1%)	9 (0.1%)	5 (0.0%)	4 (0.0%)
≥ 18 years	12 (0.2%)	4 (0.1%)	8 (0.1%)	0 (0.0%)	26 (0.1%)	6 (0.0%)	19 (0.1%)	1 (0.0%)
Unknown	667 (10.5%)	170 (2.7%)	166 (2.6%)	331 (5.2%)	4,817 (27.1%)	139 (0.8%)	161 (0.9%)	4,517 (25.4%)
Total	6,358 (100.0%)	3,067 (48.2%)	2,711 (42.6%)	580 (9.1%)	17,750 (100.0%)	6,239 (35.1%)	5,645 (31.8%)	5,866 (33.0%)

*M* Male, *F* female, *U* unknown.

### Disproportionality analysis of VAERS

For the disproportionality analysis in VAERS, we examined 17,750 safety reports related to RV vaccines and corresponding to 50,650 vaccine-reaction pairs. Reported AEFIs referring to incorrect vaccine storage, routinely laboratory tests or incorrect administration, were not considered because not pertinent to our discussion. Overall, most of the AEFIs were related to gastrointestinal disorders. The AEFIs most frequently reported and statistically significant for RV vaccines were non-serious and listed in the corresponding SPCs: pyrexia n = 1,935 events, ROR 1.17 [CI 95% 1.12–1.22], vomiting n = 1,746, ROR 3.19 [CI 95% 3.04–3.34], diarrhea n = 1672, 5.75 [CI 95% 5.48–6.04], crying n = 1,273, ROR 7.80 [CI 95% 7.38–8.25] and irritability n = 1,254, ROR 7.68 [CI 95% 7.26–8.12]. Noteworthy, the vaccine-reaction pair “RV vaccines-intussusception” showed a ROR of 27.23 [CI 95% 25.51–29.07]. Table 2 shows the top 25 AEFIs for RV vaccines by number of events. Among vaccine-reaction pairs with a higher and statistically significant ROR with RV vaccines vs. other vaccines, and a number of events greater than 25 (Table 3), we found several MedDRA Preferred Term (PT) related to the System Organ Class (SOC) “Gastrointestinal disorders” or specific for rotavirus such as: gastroenteritis rotavirus n = 96, ROR 82.49 [95% CI 63.12–107.80], rotavirus infection n = 199, ROR 81.45 [95% CI 68.64–96.65], rotavirus test positive n = 293, ROR 53.89 [95% CI 47.37–61.31] and the above-mentioned intussusception. The cut-off of 25 reports was chosen considering the total number of events and their relative frequency. A large number of AEFIs was related to hematochezia n = 843, ROR 26.27 [95% CI 24.50–28.18] and other linked events such as occult blood positive n = 189, ROR 26.06 [95% CI 22.36–30.39] and diarrhea hemorrhagic n = 106, ROR 18.87 [95% CI 15.38–23.15]. Kawasaki disease, listed in the FDA SPC of Rotarix and RotaTeq, was reported 54 times, with a ROR of 14.61 [95% CI 10.96–19.49]. Some reported events affected the respiratory system: bronchiolitis n = 27, ROR 8.80 [95% CI 5.91–13.23], choking n = 54, ROR = 7.27 [95% CI 5.51–9.59] and apnea n = 93, ROR = 6.40 [95% CI 5.19–7.88].

**Table 2:** The top 25 adverse events following immunization related to rotavirus vaccines by number of events in VAERS and VigiBase.

	VigiBase					VAERS				
	AEFI	N	ROR	CI_lower	CI_upper	Meddrapt	N	ROR	CI_lower	CI_upper
1	***Diarrhoea***	1961	9.00	8.60	9.41	Pyrexia	1935	1.17	1.12	1.22
2	***Vomiting***	1508	4.45	4.23	4.68	***Vomiting***	1746	3.19	3.04	3.34
3	***Crying***	714	1.75	1.63	1.89	***Diarrhoea***	1672	5.75	5.48	6.04
4	***Haematochezia***	610	64.02	58.65	69.89	***Crying***	1,273	7.80	7.38	8.25
5	***Abdominal pain***	572	4.26	3.92	4.62	Irritability	1,254	7.68	7.26	8.12
6	***Intussusception***	488	93.67	84.43	103.93	***Intussusception***	962	27.23	25.51	29.07
7	***Decreased appetite***	339	2.06	1.85	2.30	***Haematochezia***	843	26.27	24.50	28.18
8	***Pallor***	326	2.23	2.00	2.48	***Body temperature increased***	441	1.90	1.73	2.09
9	Discomfort	250	7.68	6.77	8.71	Screaming	414	7.32	6.64	8.08
10	Restlessness	243	2.25	1.98	2.55	Convulsion	345	2.13	1.92	2.37
11	Gastroenteritis	236	23.29	20.34	26.67	Lethargy	328	2.65	2.37	2.95
12	***Dehydration***	213	21.70	18.81	25.02	***Pallor***	327	1.58	1.42	1.76
13	***Hypotonia***	196	1.83	1.59	2.10	***Decreased appetite***	289	2.12	1.89	2.38
14	***Mucous stools***	189	43.91	37.38	51.59	***Hypotonia***	278	6.64	5.89	7.48
15	Flatulence	185	23.12	19.82	26.97	Unresponsive to stimuli	237	3.16	2.78	3.60
16	***Faeces discoloured***	182	22.51	19.28	26.30	***Dehydration***	220	6.18	5.41	7.07
17	Irritability	156	1.26	1.08	1.48	***Faeces discoloured***	215	18.39	15.98	21.16
18	Seizure	150	1.41	1.20	1.65	***Abdominal pain***	206	2.14	1.86	2.45
19	Abnormal faeces	143	26.23	21.94	31.36	***Cyanosis***	197	5.22	4.53	6.01
20	Abdominal pain upper	131	3.13	2.63	3.72	***Mucous stools***	196	23.19	19.97	26.92
21	General physical health deterioration	123	5.07	4.24	6.07	Occult blood positive	189	26.06	22.36	30.39
22	***Cyanosis***	118	2.35	1.96	2.82	Somnolence	171	2.19	1.88	2.54
23	Diarrhoea haemorrhagic	116	32.00	26.09	39.24	Dyskinesia	168	2.57	2.21	3.00
24	Hypotonic-hyporesponsive episode	110	2.33	1.93	2.82	Vomiting projectile	135	11.77	9.87	14.03
25	***Body temperature increased***	108	1.22	1.01	1.48	Diet refusal	134	7.33	6.16	8.71

*AEFI* Adverse Events Following Immunization, *ROR* reporting odds ratio, *CI* 95% confidence interval, *CI_lower* lower limit of the confidence interval, *CI_upper* upper limit of the confidence interval.

**Table 3:** Most‐reported Adverse Events Following Immunization and corresponding Reporting Odds Ratio (ROR) for rotavirus vaccines compared to other vaccines in the databases.

VigiBase					VAERS				
AEFI	N^a^	ROR	CI_lower	CI_upper	AEFI	N^a^	ROR	CI_lower	CI_upper
Rotavirus test positive	419	868.85	670.48	1,125.91	Gastroenteritis rotavirus	96	82.49	63.12	107.80
Gastroenteritis rotavirus	1,104	858.41	762.50	966.38	Rotavirus infection	199	81.45	68.64	96.65
Rotavirus infection	752	791.30	678.88	922.34	Rotavirus test positive	293	53.89	47.37	61.31
Viral test positive	60	489.91	197.90	1,212.79	Intussusception	962	27.23	25.51	29.07
Intussusception	488	93.67	84.43	103.93	Haematochezia	843	26.27	24.50	28.18
Haematochezia	610	64.02	58.65	69.89	Infantile spitting up	121	24.52	20.20	29.78
Mucous stools	189	43.91	37.38	51.59	Mucous stools	196	23.19	19.97	26.92
Frequent bowel movements	76	41.40	31.65	54.14	Intestinal obstruction	26	20.87	13.34	32.65
Infantile spitting up	82	38.66	29.98	49.85	Diarrhoea haemorrhagic	106	18.87	15.38	23.15
Diarrhoea haemorrhagic	116	32.00	26.09	39.24	Faeces discoloured	215	18.39	15.98	21.16
Enteritis	49	28.56	20.60	39.60	Occult blood	37	18.06	12.59	25.90
Fluid intake reduced	88	28.03	22.17	35.43	Sudden infant death syndrome	54	17.46	13.04	23.39
Abnormal faeces	143	26.23	21.94	31.36	Feeding disorder of infancy or early childhood	42	16.33	11.71	22.79
Gastroenteritis	236	23.29	20.34	26.67	Fontanelle bulging	45	16.23	11.78	22.37
Flatulence	185	23.12	19.82	26.97	Abnormal faeces	75	15.06	11.82	19.19
Faeces discoloured	182	22.51	19.28	26.30	Kawasaki's disease	54	14.61	10.96	19.49
Regurgitation	49	22.21	16.19	30.48	Hypotonic-hyporesponsive episode	33	12.44	8.59	18.03
Dehydration	213	21.70	18.81	25.02	Frequent bowel movements	57	12.03	9.12	15.85
Weight gain poor	30	21.58	14.24	32.71	Vomiting projectile	135	11.77	9.87	14.03
Metabolic acidosis	29	21.50	14.08	32.83	Gastroenteritis	49	11.29	8.38	15.22
Choking	34	18.91	12.92	27.67	Infantile spasms	33	10.81	7.49	15.60
Rectal haemorrhage	38	15.76	11.09	22.39	Haematemesis	36	9.63	6.80	13.64
Hypophagia	60	13.36	10.19	17.52	Flatulence	91	9.48	7.66	11.74
Faeces soft	27	13.21	8.71	20.03	Grunting	34	9.10	6.36	13.00
Vaccination failure	1527	9.24	8.78	9.72	Bronchiolitis	27	8.84	5.91	13.23
Diarrhoea	1961	9.00	8.60	9.41	Crying	1,273	7.80	7.38	8.25
Vomiting projectile	39	8.37	6.01	11.66	Breath holding	35	7.71	5.44	10.92
Discomfort	250	7.68	6.77	8.71	Irritability	1,254	7.68	7.26	8.12
Floppy infant	26	7.66	5.09	11.52	Diet refusal	134	7.33	6.16	8.71
Gastrooesophageal reflux disease	32	6.52	4.54	9.38	Screaming	414	7.32	6.64	8.08
Pharyngeal erythema	42	5.97	4.37	8.18	Choking	54	7.27	5.51	9.59
Apparent life threatening event	26	5.63	3.76	8.41	Hypotonia	278	6.64	5.89	7.48
Kawasaki's disease	33	5.57	3.90	7.94	Apnoea	93	6.40	5.19	7.88
Constipation	105	5.08	4.18	6.17	Hypophagia	66	6.30	4.91	8.07
General physical health deterioration	123	5.07	4.24	6.07	Dehydration	220	6.18	5.41	7.07
Poor feeding infant	38	4.94	3.56	6.87	Diarrhoea	1672	5.75	5.48	6.04
Livedo reticularis	26	4.54	3.05	6.76	Opisthotonus	39	5.55	4.01	7.68
Vomiting	1508	4.45	4.23	4.68	Gastrooesophageal reflux disease	62	5.30	4.11	6.85
Abdominal pain	572	4.26	3.92	4.62	Foaming at mouth	28	5.28	3.59	7.76
Respiratory arrest	30	4.24	2.93	6.14	Emotional distress	102	5.23	4.29	6.37
Underdose	57	4.24	3.25	5.53	Cyanosis	197	5.22	4.53	6.01
Infantile spasms	26	4.05	2.72	6.02	Listless	53	5.07	3.85	6.69
Abdominal distension	58	4.03	3.10	5.25	Abdominal distension	54	4.57	3.48	6.01
Upper respiratory tract infection	46	3.91	2.91	5.25	Staring	132	4.43	3.73	5.27
Weight decreased	74	3.88	3.08	4.89	Constipation	76	4.37	3.47	5.50
Retching	41	3.86	2.82	5.28	Depressed level of consciousness	51	3.27	2.47	4.33
Screaming	79	3.78	3.02	4.73	Vomiting	1746	3.19	3.04	3.34
Viral infection	49	3.58	2.69	4.76	Gaze palsy	123	3.17	2.65	3.79
Apnoea	96	3.29	2.69	4.03	Unresponsive to stimuli	237	3.16	2.78	3.60
Eye movement disorder	45	3.14	2.33	4.22	Posture abnormal	64	3.15	2.45	4.04
Abdominal pain upper	131	3.13	2.63	3.72	Inappropriate schedule of drug administration	890	3.06	2.87	3.27
Sepsis	26	3.04	2.05	4.51	Eye movement disorder	77	3.00	2.40	3.77
Unresponsive to stimuli	60	3.01	2.33	3.89	Retching	56	2.99	2.29	3.90
Listless	102	2.82	2.32	3.43	Otitis media	27	2.69	1.83	3.95
Drug administered to patient of inappropriate age	59	2.46	1.90	3.18	Secondary transmission	26	2.65	1.79	3.93
Cyanosis	118	2.35	1.96	2.82	Lethargy	328	2.65	2.37	2.95
Hypotonic-hyporesponsive episode	110	2.33	1.93	2.82	Eczema	41	2.64	1.93	3.60
Infection	45	2.33	1.73	3.13	Dyskinesia	168	2.57	2.21	3.00
Apathy	66	2.29	1.80	2.93	Hypersomnia	78	2.51	2.00	3.14
Restlessness	243	2.25	1.98	2.55	Seizure like phenomena	41	2.25	1.65	3.07
Pallor	326	2.23	2.00	2.48	Abnormal behaviour	96	2.24	1.83	2.74
Incorrect route of drug administration	43	2.21	1.63	2.99	Somnolence	171	2.19	1.88	2.54
Decreased appetite	339	2.06	1.85	2.30	Abdominal pain	206	2.14	1.86	2.45
Abdominal discomfort	28	2.01	1.38	2.93	Convulsion	345	2.13	1.92	2.37

*AEFI* adverse events following immunization, *ROR* reporting odds ratio, *CI* 95% confidence interval, *CI_lower* lower limit of the confidence interval, *CI_upper* upper limit of the confidence interval.

^a^Only events reported more than 25 times were included in the table.

### Disproportionality analysis of VigiBase

For the disproportionality analysis, we analyzed 6,358 safety reports related to RV vaccines and corresponding to 23,059 vaccine-reaction pairs in VigiBase. The AEFIs most frequently reported and statistically significant for RV vaccines were non-serious and listed in the corresponding SPCs: diarrhea n = 1,961 events, ROR 9.00 [95% CI 8.60–9.41], vomiting n = 1,508, ROR 4.45 [CI 95% 4.23–4.68], crying n = 714, ROR 1.75 [CI 95% 1.63–1.89], hematochezia n = 610, ROR 64.02 [CI 95% 58.65–69.89] and abdominal pain n = 572, ROR 4.26 [CI 95% 3.92–4.62]. In VigiBase as well as in VAERS, the vaccine-reaction pair “RV vaccines-intussusception” showed a ROR of 93.67 [95% CI 84.43–103.93]. Table 2 shows the top 25 AEFI for RV vaccines in term of number of events. The most frequently reported AEFIs with RV vaccines were gastrointestinal disorders. Many AEFIs were specific for RV vaccines: gastroenteritis rotavirus n = 1,104, rotavirus infection n = 752, rotavirus test positive n = 419, viral test positive n = 60. Other frequently reported AEFIs were: pallor n = 326, ROR 2.06 [95% CI 1.85–2.30], discomfort n = 250, ROR 7.68 [95% CI 6.77–8.71], restlessness n = 243, ROR 2.25 [95% CI 1.98–2.55], hypotonia n = 196, ROR 1.83 [95% CI 1.59–2.10] and irritability n = 156, ROR 1.26 [95% CI 1.08–1.48]. Among the vaccine-reaction pairs with a higher and statistically significant ROR vs. other vaccines (Table 3) we found some not related to gastrointestinal disorders such as chocking n = 34, ROR 18.91 [95% CI 12.92–27.67], Kawasaki’s disease n = 33, ROR 5.57 [95% CI 3.90–7.94], general physical health deterioration n = 123, ROR 5.07 [95% CI 4.24–6.07], livedo reticularis n = 26, ROR 4.54 [95% CI 3.05–6.76], retching n = 41, ROR 3.86 [95% CI 2.82–5.28], screaming n = 79, ROR 3.78 [95% CI 3.02–4.73] and apnea n = 96, ROR 3.29 [95% CI 2.69–4.03].

## Discussion

The post-marketing evaluation of vaccines safety profile is a mainstay in the vaccines life cycle. Vaccines represent one of the greatest medical achievements but their recognized effectiveness often runs the risk of being overshadowed by conjectures relating to their safety profile.

Spontaneous reporting systems allow retrieving real-life safety data regarding medicinal products without the restricted inclusion criteria of clinical trials. In recent years, stratified pharmacovigilance studies on pediatric population have received great attention. A stratification analysis is currently underway to investigate signal detection in vulnerable population including pediatrics^23^. However, pharmacovigilance studies based on spontaneous reporting may have some limitations that need to be acknowledged^24^. First of all, the lack of a comparison unvaccinated group does not allow to calculate any incidence rate. The same is also true for the absence of information about the background rates of natural events described in the safety reports. In addition, the data reported in VAERS as well as in VigiBase, may be incomplete and inaccurate, also including the incorrect attribution of the severity criterion. In fact, the reports may not only be health care professionals but also citizens, patients and health insurances. Another limitation is represented by the underreporting (i.e. lack of reports for all ADRs that actually occur), that contribute to underestimate the number of ADRs occurring^25^. The AEFIs already reported in the SPCs can be reported more easily than the unknown ones, as the reporter finds confirmation of what he wants to report. Also selective reporting influences the studies with pharmacovigilance databases: modest and expected AEFIs tend to be reported less frequently. Also to note that none of the potential co-administered vaccines has been considered. Therefore differentiating effects secondary to rotavirus as compared to other concomitantly administered vaccines is not possible. Also Reporting Odds Ratio has potential inherent biases as it does not allow to establish any certain causality relation between vaccine and AEFI, but highlights an association between the drug and the adverse event and the potential disproportionality. Also different reporting trends over time and across specific categories of vaccines for certain type of AEFI, as it happens in case of safety warnings issued by regulatory agency, may influence the spontaneous reporting in pharmacovigilance. Lastly, we do not considered different time windows in our analysis. Especially with regards to intussusception, it would be useful to know the time elapsed from the vaccine administration to the onset of the event but rarely these data are accurately reported in spontaneous reporting. This may be the aim of others pharmacoepidemiological studies. Considering strengths and limitations all together, it is clear that the limitations do not represent an obstacle to pharmacovigilance studies but only that they have to be taken into account in the interpretation of the results. To date, there are no other sources that allow obtaining so much safety data such as pharmacovigilance databases.

Overall, our data shows that the total number of safety reports increased over the years for both the RV vaccines and databases. RotaTeq spontaneous reporting in VAERS followed a different trend compared to Rotarix in both the database and to RotaTeq in VAERS. This vaccine was recommended by the Advisory Committee on Immunization Practices (ACIP) for routine vaccination of US infant in 2006. The subsequent peak in 2007 appears to reflect the Weber effect, i.e. an epidemiologic phenomenon stating that the number of reported adverse reactions rises until approximately the middle to end of the second year of marketing, peaks, and then declines^26^. In 2013, the FDA approved the inclusion of new safety information on the risk of intussusception in the package insert and patient package insert of RotaTeq. This may explain the second peak between 2013 and 2014. In addition, the different health policies in force in several countries and the diverse attitude towards spontaneous reporting could have influence these trends. In Europe, most of reporters are health care professionals, while in the US citizens contribute largely. Moreover, spontaneous reporting in the US can be affected by the health insurance system in force. In the US, there are specific programs to reward those injured by vaccinations, which can increase the number of reports from citizens. One of these is the National Vaccine Injury Compensation Program, that for RV vaccines reports that only 40 rewards has been achieved during the years 2007–2017 in front of 107,678,219 doses of RV vaccines distributed^27^. Considering the distributed doses and the number of serious reports (2,953) in VAERS during the same period, a rate of three reports per 100,000 doses rounding up emerged from our research.

Currently for infants in the US, RotaTeq is given in 3 doses at 2, 4 and 6 months, and Rotarix is given in 2 doses at 2 and 4 months^28^. Also in Europe, the first dose of RV vaccines is recommended at 2 months, except for Czech Republic, Germany, Poland and Norway where it is suggested at 6 weeks^29^. The number of doses depends on the vaccine brand used. Children should receive all doses of RV vaccine before they turn 8 months old. Our data from VAERS are in line with this information, as we observed that only 1.2% of safety reports involved patients aged ≥ 1 years. Although in Europe RV vaccines are not indicated for the population ≥ 8 months, we found a number ten times higher of safety reports in VigiBase for the age class ≥ 1 year or probably related to misreporting. RV is the leading cause of severe, dehydrating diarrhea in children less than 5 years old^5^, in some situations it may happen that for prevention the vaccine is administered off-label by age. No significant difference emerged with regard to sex.

Overall, our analysis confirms the favorable safety profile of RV vaccines. To not forget that these vaccines contribute to reduce RV-associated mortality^5^. Fourteen out of the 25 most reported AEFIs were present in both databases. Most of them were mild AEFIs already acknowledged in the SPCs of the corresponding vaccine, such as diarrhea, vomiting, abdominal pain, hematochezia, decreased appetite, mucous stool and dehydration. This evidence confirms the reliability of our analysis, which highlighted that the most frequent AEFIs corresponded to those reported in SPCs. The majority of AEFIs were related to gastrointestinal disorders.

RV vaccines are live attenuated oral vaccines and to produce an immune response, they must replicate in the vaccinated person. In certain situation, this may cause the same symptoms, but in a much milder form, which would cause the actual viral infection^30^. Noteworthy, among gastrointestinal disorders, intussusception appeared as one of the most reported AEFIs. To note that we do not considered different time windows in our analysis. The evaluation of the time elapsed from the vaccine administration to the onset of intussusception is important to better understand the possible causal relation but this information is rarely reported in spontaneous safety reports correctly. The possible association between RV vaccines and intussusception started to be discussed in 1999 after Rotashield withdrawal. To date, many cases of intussusception after RV vaccination have been evaluated^15,31–34^. Several articles in literature tried to estimate the risk of intussusception in the vaccinated and unvaccinated groups giving important results^35^. Even though a correlation between RV vaccination and intussusception has been confirmed, this doesn’t change the evaluation of the benefit/risk profile that remains favorable. To better understand the relationship of intussusception to RV vaccines, research is needed to further delineate the potential etiologies and mechanisms of intussusception^36–39^. Remarkably, we also observed a potential association between RV vaccines and Kawasaki’s disease (KD) in both the two databases analyzed. In VAERS this vaccine-event pair showed a ROR of 14.61 [95% CI 10.96–19.49]. While KD is acknowledged as a possible AEFI in the FDA SPC of both RV vaccines, it is not listed in the EMA SPC of Rotarix. Previous findings from pre-licensure trials of RotaTeq suggested a possible association between RV vaccines and KD. In 2008, the US Food and Drug Administration amended the product information for the United States in order to record any cases of this AEFI^40^. The SPCs in US and Europe should be harmonized accordingly. In addition, several AEFIs related to respiratory apparatus showed high reporting frequency in our analysis. Among these, we highlighted chocking, pharyngeal erythema, respiratory arrest, upper respiratory tract infection, apnea, breath holding and bronchiolitis. A recent study based on VAERS analysis focused on safety reports of lower respiratory tract infection (LRTI) after RV vaccination^41^. They concluded that despite limitations, no significant differences in reports of LRTI following RV vaccinations was found in VAERS. To date, only cough, runny nose and bronchiolitis are listed in the SPCs with slight difference between EMA and FDA, highlighting once again the need of an alignment. About chocking, more than an AEFI it seems related to the route of administration. WHO guideline report to never place the tube of the vaccine into the center of the mouth to prevent the risk of choking^42^. It is important to be aware of possible maladministration of these vaccines as reported by Hibbs et al.^43^. Other events not listed in the SPC that will deserve future investigations are: fontanelle bulging, hypotonic-hyporesponsive episode, livedo reticularis, infantile spasms, opisthotonus and seizure like phenomena. All these showed a high statistically significant ROR in our research. The correct comprehension of RV vaccine safety profiles is important to allow a high vaccination coverage. The Global Health Observatory (GHO) data report that RV vaccines were introduced in 91 countries by the end of 2017, and global coverage was estimated at 28%. In the US, the vaccination coverage rose from 44% in 2009 to 73% in 2017. Same data are reported also for Europe^44^, with great differences from one country to another. To date, 14 countries in Europe lack national government recommendations for RV vaccines in children^45^. The lack of recommendations may influence public perception towards vaccinations.

## Conclusion

Overall, our data shows that most of the reported AEFIs are related to gastrointestinal system and they are listed in the Summary of Product Characteristics (SPCs) of the corresponding vaccine. It would be important to describe more in depth all the single possible events related to the gastrointestinal system, in order to detect potential intussusception immediately. There also remains the need to investigate new potential safety signals arose from this analysis, in order to complete the description of the AEFIs in the SPCs. Rotavirus vaccines have decreased severe rotavirus gastroenteritis in many countries and RV-associated mortality and to date their safety profile is in line with the reported events in the SPCs.
